# Loss to follow-up from antiretroviral therapy clinics: A systematic review and meta-analysis of published studies in South Africa from 2011 to 2015

**DOI:** 10.4102/sajhivmed.v20i1.984

**Published:** 2019-12-18

**Authors:** Samantha Kaplan, Katleho S. Nteso, Nathan Ford, Andrew Boulle, Graeme Meintjes

**Affiliations:** 1 Department of Internal Medicine, University of Washington, Seattle, United States; 2 Medical Care Development International, Maseru, Lesotho, South Africa; 3 School of Public Health and Family Medicine, University of Cape Town, Cape Town, South Africa; 4 Centre for Infectious Disease Epidemiology and Research, School of Public Health and Family Medicine, University of Cape Town, Cape Town, South Africa; 5 Institute of Infectious Disease and Molecular Medicine, Faculty of Health Sciences, University of Cape Town, Cape Town, South Africa

**Keywords:** HIV, antiretroviral therapy (ART), loss to follow-up, disengagement, South Africa

## Abstract

**Background:**

South Africa has the largest antiretroviral therapy (ART) programme in the world. To optimise programme outcomes, it is critical that patients are retained in care and that retention is accurately measured.

**Objectives:**

To identify all studies published in South Africa from 2011 to 2015 that used loss to follow-up (LTFU) as an indicator or outcome to describe the variation in definitions and to estimate the proportion of patients lost to care across studies.

**Method:**

All studies published between 01 January 2011 and October 2015 that included loss to follow-up or default from ART care in a South African cohort were included by use of a broad search strategy across multiple databases. To be included, the cohort had to include any patient ART data, including follow-up time, from 01 January 2010. Two authors, working independently, extracted data and assessed risk of bias from all manuscripts. Meta-analysis was performed for studies stratified by the same loss to follow-up definition.

**Results:**

Forty-eight adult, 15 paediatric and 4 pregnant cohorts were included. Median cohort size was 3737; follow-up time ranged from 9 weeks to 5 years. Meta-analysis did not reveal an important difference in LTFU estimates in adult cohorts at 1 year between loss to follow-up defined as 3 months (11.0%, *n* = 4; 95% CI 10.7% – 11.2%) compared with 6 months (12.0%, *n* = 4; 95% CI 11.8% – 12.2%). Only two cohorts reported reliable LTFU estimates at 5 years: this was 25.1% (95% CI 24.8% – 25.4%).

**Conclusion:**

South Africa should standardise a LTFU definition. This would aid in monitoring and evaluation of ART programmes, with the broader goal of improving patient outcomes.

## Introduction

As of 2016, over 7 million people in South Africa were living with human immunodeficiency virus (HIV), of which 56% were receiving antiretroviral therapy (ART). This represents the largest ART programme in the world^1^: South Africa’s ART population accounts for 20% of people on ART globally, and the country instituted updated national guidelines in 2016 to offer ART to all patients with HIV. Because of this rapid upscaling and increasing number of patients eligible for and starting lifelong ART, a focus on retention in ART care has become even more important. According to a review in South Africa from 2008 to 2013, only ± 67% of patients who initiated ART remained in care after 4 years, and 40% of those who were lost were attributed to known deaths.^2^

Since the first availability of HIV treatment, studies have reported findings on retention after ART initiation, usually as an indicator of programme effectiveness. There has not been a definitive temporal trend: some studies have concluded that loss to follow-up (LTFU) proportions are decreasing over time, but others concluded that rates have increased as the epidemic has grown, coinciding with the increase in numbers of patients enrolled at health centres that serve ART patients.^3,4,5,6^ More recent studies have supported the notion that with increasing CD4 thresholds for ART initiation and the adoption of ‘test and treat’, LTFU rates are increasing.^7,8,9^ However, a large part of the variation in reported outcomes is because of the lack of standardisation of definitions of LTFU and retention, as well as the bias in reporting interruptions in care.^10,11,12^ If patient retention is to be used as a key indicator of ART programme effectiveness, there should be a standardised definition of LTFU so that ART programmes can be more accurately compared within and between countries.

The aims of this systematic review were to identify all studies published in South Africa from 2011 to 2015 that used LTFU as an indicator or outcome, to describe the variation and diversity of definitions as justification for establishing a single standardised definition going forward, to summarise the findings using meta-analysis and to provide suggestions for ways to use LTFU as an indicator in a standardised fashion. Reporting standards have evolved since ART began to be provided in the South African public sector in 2004, as have treatment guidelines. This review focuses on the 5 years between 2011 and 2015, when there were more stringent requirements to start ART; those who started had lower CD4 counts. Since 2015, universal test and treat has been adopted in South Africa and even more patients have been enrolled in ART. Based on the findings of this review, we provide suggestions for ways to use ‘LTFU’ as an indicator in a standardised fashion with an increasing population of patients on ART.

## Methods

This systematic review was designed, conducted and reported in accordance with the Preferred Reporting Items for Systematic Reviews (PRISMA) statement.^13^ The protocol was registered on PROSPERO International Prospective Register of Systematic Reviews as #CRD42015026466 (http://www.crd.york.ac.uk/PROSPERO).

### Eligibility criteria

All studies found in the search engines and published between 01 January 2011 and October 2015 that included loss to follow-up or default from ART care as an indicator or outcome in a South African ART cohort were included, even if that cohort was part of an interventional trial. If the cohort was composed of a mix of pre-ART and ART patients, we only reported outcomes for ART patients; cohorts that were not disaggregated were excluded. To be included, the cohort analysed had to (1) be published between January 2011 and October 2015 and (2) report any patient ART data, including follow-up, from 01 January 2010; however, initial data could have been collected before this time point. These criteria were enacted so as not to include older data in the analysis if a manuscript was not published until much later. Both adult and paediatric studies were included. If the article was multinational, it was included only if the data were disaggregated and reported South African data separately. Systematic review articles were excluded, but their citation lists were reviewed for further eligible articles. Modelling studies were excluded. Interventional studies were included if they reported loss to follow-up, and risk of bias was assessed on the observational component of these studies.

### Search strategy and information sources

**FIGURE 1:**
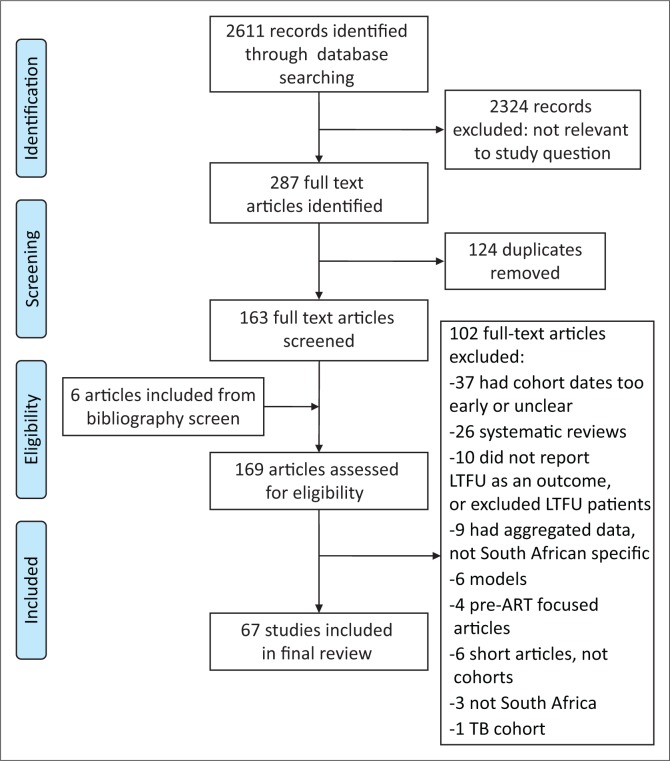
Flowchart of article selection for inclusion in the study.

By use of a broad search strategy (Appendix 1), one investigator (S.K.) worked independently to search MEDLINE via PubMed, EMBASE via Scopus, Web of Science, CINAHL and Africa-Wide databases from 01 January 2011 to date of search. PubMed was searched on 05 October 2015, Scopus and Africa-Wide on 07 October 2015, Web of Science on 14 October 2015 and CINAHL on 15 October 2015. Information specialists at the University of Cape Town Medical Library assisted with the literature search process. After obtaining lists of abstracts meeting the search criteria from each database, two investigators (S.K. and K.S.N.) reviewed the abstracts independently in duplicate and met to achieve consensus on final inclusions of full-text review. S.K. and K.S.N. supplemented database searches by screening bibliographies of all full-text articles screened for the review. Figure 1 details the article selection process.

### Data extraction

Data were extracted independently in duplicate (S.K., K.S.N.) using a standardised extraction form. Data collection forms were crosschecked by both reviewers, and the reviewers discussed discrepancies, with differences resolved by a third investigator (G.M.). Data were extracted on study design, dates, description and context of intervention, if applicable, participant characteristics (age, baseline CD4 count), programme characteristics (cohort size, number of clinics, eligibility criteria), length of follow-up, definition of outcomes, outcomes, missing data and study limitations.

Primary outcomes were LTFU and mortality. If a study reported these outcomes by sub-cohort instead of aggregate, then outcomes by sub-cohort were recorded. LTFU outcomes at 1 and 5 years were collected, when available, for meta-analysis. If raw numbers were not readily available from the text, the reviewers calculated it from the available text or figures and agreed on the numerator and denominator. If a study included both pre-ART and ART patients, the statistics were only calculated for ART patients; pre-ART patients were excluded. If the study did not provide a definition for LTFU or default, or had another issue needing clarification, S.K. contacted the corresponding author by email.

### Assessment of study quality

Study quality and risk of bias were assessed by evaluating the selection of the cohort, ascertainment of outcomes, length of follow-up and the presence of missing data using a modified set of criteria based on the Newcastle-Ottawa domains^14^ (Appendix 2).

### Meta-analysis

For a study to be included in the meta-analysis, it had to have raw data available for a total number of patients LTFU at 12 months and/or 5 years of ART. Some of these studies had overlapping data in that the data were collected from the same clinic population with some overlapping time periods. If it was not clear if the data were overlapping, the reviewers emailed the manuscript authors for verification. If the data did overlap, the reviewers selected the most recent cohort with the largest amount of data available.

Point estimates and 95% confidence intervals were calculated for the proportion of people LTFU and data were pooled following transformation using random-effects meta-analysis. Differences in the definitions of LTFU (3 months vs. 6 months) and between patient groups (adults vs. children vs. pregnancy) were assessed through pre-planned subgroup analyses. Point estimates and 95% confidence intervals were displayed visually on a forest plot to visually assess heterogeneity. All data were analysed with STATA version 14.0.

### Ethical considerations

This article followed all ethical standards for research without direct contact with human or animal subjects.

## Results

During the primary database search, 2611 abstract citations were identified and 2324 were excluded. After removing duplicates, 163 full-text articles were screened for inclusion and six additional articles were included from a bibliography screen of these articles; 67 articles were included in the final review (Figure 1).

Of the 67 eligible studies, 48 were adult cohorts, 15 were paediatric cohorts and four were focussed on pregnant women; 57 studies included study or follow-up time prior to 2010. Mean cohort size was 10,711; median was 3737. Only six studies were interventional; the rest were observational. Seven studies (10%) utilised research data; the remainder of the studies utilised routinely collected data from ART clinics. Follow-up time ranged from 9 weeks to 5 years, with a large variation in how this was calculated. Forty-six cohorts were solely in primary care clinics, while four were solely in clinics located in hospitals and 15 were in both primary care and hospital clinics. Forty-five cohorts (67%) were in urban settings, 7 (10%) were in rural settings and 13 (19%) were in both urban and rural settings; 2 (3%) studies were missing this information. Twenty-seven cohorts (40%) were in the Gauteng province, 11 (16%) in the Western Cape, 7 in KwaZulu-Natal (10%), 1 (1%) in the Free State, 1 (1%) in Limpopo and 2 (3%) did not include the information; 18 studies (27%) included data from multiple provinces, which included Gauteng, Western Cape, KwaZulu-Natal, Mpumalanga, Eastern Cape, Limpopo, Free State and North West provinces.

For the 33 adult cohorts that reported age in aggregate, the median age was 35.8 years, and for the 32 adult cohorts reporting CD4 count, the median baseline CD4 was 121 cells/µL. Among the paediatric cohorts, the median age was 4.2 years at ART initiation, and the median aggregate CD4 percentage was 12.5%. In the four pregnancy cohorts, the median age was 28 years (*n* = 3 cohorts reporting), and the median CD4 estimate was 239 cells/µL. In terms of definitions, 24 adult cohorts defined LTFU as 3 months without a clinic visit, 18 adult cohorts defined LTFU as 6 months without a clinic visit and 6 adult cohorts had other definitions, such as a different length of time without a clinic visit or no definition of LTFU included in the manuscript text. Of the paediatric cohorts, 2 cohorts defined LTFU as 3 months without a clinic visit, 6 cohorts defined as 6 months without a clinic visit and 7 cohorts had other definitions. Among the pregnancy cohorts, one defined LTFU as 3 months without a clinic visit and the other three had other definitions (Online Appendix 1 and 2^15,16,17,18,19,20,21,22,23,24,25,26,27,28,29,30,31,32,33,34,35,36,37,38,39,40,41,42,43,44,45,46,47,48,49,50,51,52,53,54,55,56,57,58,59,60,61,62,63,64,65,66,67,68,69,70,71,72,73,74,75,76,77,78,79,80,81^).

Of the 96 cohorts reporting mortality, encompassed within the 67 studies, the median mortality estimate was 7.9% (interquartile range [IQR] 4.1% – 11.4%; range 0% – 26%); range of time for reporting was 3 months to 5 years. There was significant variability in how these estimates were calculated; some were raw data reported at a certain endpoint; some were estimated using statistical methods; and some studies utilised linkage of patients to the national death registry. Of those 17 estimates in the lowest quartile (< 4% mortality), all had *n* < 5000; nine (53%) had *n* < 1000. Ten of these cohorts (41%) estimated mortality at < 2 years of follow-up, 6 (35%) did not standardise mortality estimates and the remaining 4 (24%) were paediatric studies with longer follow-up. Of the 16 estimates in the highest quartile (> 11.4% mortality), 10 cohorts (63%) had *n* > 2000, 5 cohorts (31%) had *n* < 1000, of which 3 were paediatric studies. Only five studies (29%) standardised a timeframe for mortality estimates, ranging from 1 to 4 years. Two (12.5%) were interventional studies. Of the total 19 cohorts reporting mortality at 1 year, the median mortality was 9.6% (range 3.8% – 17.4%). Only three cohorts reported mortality at 5 years with a median of 9.0% (range 8.6% – 10.6%) (Online Appendix 2).

Of the 101 cohorts reporting LTFU, encompassed within the 67 studies, the median LTFU estimate was 12.8% (IQR 7.9% – 22.0%; range 0.2% – 43.1%); range of time for reporting was 3 months to 5 years. Of those 14 estimates in the lowest quartile (< 7.9% LTFU), four cohorts (28.6%) had *n* < 2000; five (36.0%) had *n* < 5000. Eight (57%) were paediatric studies, and 1 (7%) was an adult interventional study. Half did not standardise their LTFU estimate; the other half estimated at 3 years or under. Of the 20 estimates in the highest quartile (> 22% LTFU), 12 studies (60%) had *n* < 1000, and 4 (20%) had *n* < 100; 2 (10%) studies were paediatric cohorts, 4 (20%) studies were pregnancy cohorts and 1 (5%) study was an interventional pregnancy cohort. The timeline for estimating LTFU ranged from 6 months to 3 years, with 12 cohorts (60%) not reporting a standardised timeframe (Online Appendix 2).

The vast majority of studies had reliable data collection (99%), an independent assessment of outcome (99%), and reported mortality (96%); 75% of cohorts were deemed definitely representative of the population, with only 9% definitely not or unclear. In terms of follow-up, seven studies (10%) had follow-up of greater than 3 years; the majority of studies had follow-up lengths between 1 and 3 years (*n* = 49; 73%); seven studies (10%) had follow-up shorter than 1 year, and four studies (6.0%) had follow-up of unclear length; 54% of studies had complete data, while 30% of studies were missing < 10% of data related to our primary outcomes, 10% of studies were missing > 10% of data related to our primary outcomes and 6% of studies did not state anything about the missing data in the manuscript.

### Meta-analysis

**FIGURE 2:**
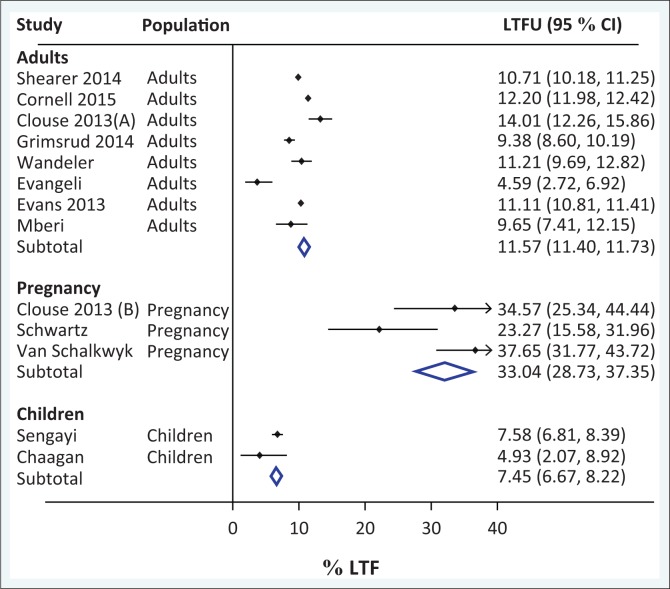
Meta-analysis of proportion of patients loss to follow-up at 1 year, by study population.

**FIGURE 3:**
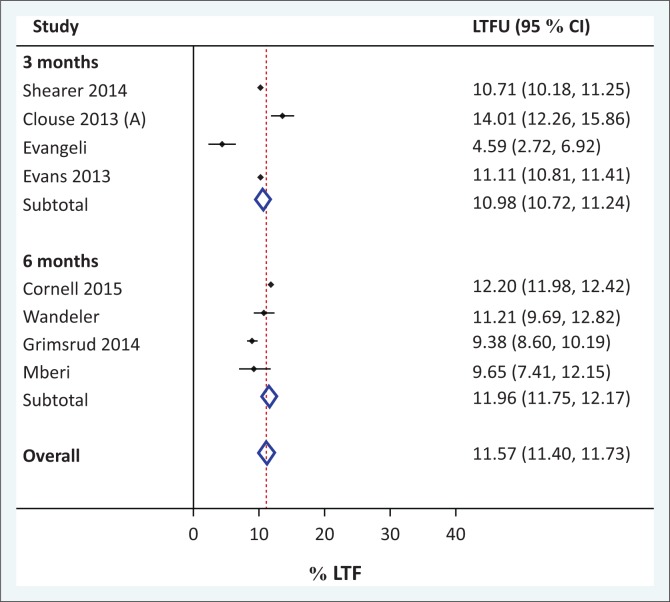
Meta-analysis of proportion of patients loss to follow-up at 1 year, by loss to follow-up definition (3 months vs. 6 months).

Aggregate LTFU estimates at 1 year were 11.6 (95% CI 11.4% – 11.7%) for eight representative adult cohorts, 33.0% for three pregnancy cohorts (95% CI 28.7% – 37.4%) and 7.5% (95% CI 6.7% – 8.2%) for two paediatric cohorts (Figure 2). The same analysis was performed after taking definitions of LTFU into account: LTFU estimates at 1 year for adult cohorts did not substantially differ between the 3-month definition (11.0%, *n* = 4; 95% CI 10.7% – 11.2%)^15,16,17,18^ and 6-month definition (12.0%, *n* = 4; 95% CI 11.8% – 12.2%) (Figure 3).^19,20,21,22,81^ There were only two cohorts that reported reliable LTFU estimates at 5 years; in aggregate, this was 25.1% (95% CI 24.8% – 25.4%).^22,81^ Statistical heterogeneity of LTFU was quite large as anticipated, as estimated by visual inspection of the forest plots. Table 1 summarises the characteristics and key figures from each study included in the meta-analysis.^15,16,17,18,19,20,21,22,34,66,70,77,79,81^

**TABLE 1:** Characteristics of studies included in the meta analysis.

First author, ref	Population	LTFU definition	Cohort size	Study type	Dates of cohort	Rural versus urban	Province	Age, in years unless otherwise reported		Baseline CD4, cells/mm^3^ unless otherwise reported		# LTFU (12 months)	# LTFU (5 years)
								Median	IQR	Median	IQR		
Shearer^15^	Adults	3 months	12 840	Obs	2004–2013	Urban	Gauteng	36.9	31.6–43.5	98	36–169	1375	-
Clouse^16^	Adults	3 months	1430	Obs	2010–2012	Urban	Gauteng	34.4	29.3–41.2	195	103–266	200	-
Evangeli^17^	Adults	3 months	380	Obs	2009–2013	Rural	KwaZulu-Natal	35	29–43	133	76–175	17	-
Evans^18^	Adults	3 months	42 427	Obs	2004–2011	Mixed	Gauteng, Mpumalanga	NR	NR	NR	NR	4713	-
Cornell^19^	Adults	6 months	83 566	Obs	2004–2013	Mixed	Western Cape, Gauteng, KwaZulu-Natal	NR	NR	125	56–192	10 195	-
Wandeler^20^	Adults	6 months	1556	Obs	2007–2010	Urban	Western Cape, Gauteng	NR	NR	NR	NR	174	-
Fatti^22^	Adults	6 months	90 071	Obs	2004–2011	Mixed	Western Cape, Eastern Cape, KwaZulu-Natal, Mpumalanga	NR	NR	NR	NR	-	22 604
Grimsrud^81^	Adults	6 months	5154	Obs	2002–2012	Urban	Western Cape	33.8	28.9–40.2	121	60–184	483	1081
Mberi^21^	Adults	6 months	595	Obs	2004–2012	Urban	Gauteng	35.9	30.1–43.5	NR	NR	57	-
Sengayi^66^	Children	6 months	4266	Obs	2004–2012	Urban	Gauteng	4.2	1.4–7.4	mean 14.5%	s.d. 9.3	323	-
Chhagan^70^	Children	Other	151	Obs	2006–2011	Urban	KwaZulu-Natal	61.3 months	29.6–90.1	12.6%	8.0–19.1	7	-
Van Schalkwyk^77^	Pregnancy	3 months	250	Obs	2008–2010	Urban	Western Cape	28	25–32	176	112–218	94	-
Clouse^34^	Pregnancy	Other	93	Obs	2010–2011	Urban	Gauteng	27	24–31	244	171–299	32	-
Schwartz^79^	Pregnancy	Other	100	Int	2013–2014	Urban	Gauteng	NR	NR	434	297–515	23	-

LTFU, lost to follow-up; Obs, observational study; Int, interventional study; NR, not reported; s.d., standard deviation; IQR, interquartile range.

## Discussion

Among the 67 studies reporting LTFU from ART care in South Africa that met our inclusion criteria, LTFU definitions and estimation methodologies were not standardised. Our meta-analysis did not indicate any important difference in loss to follow-up estimates for those studies when using 3-month versus 6-month definitions.

The median non-standardised LTFU estimate for all studies (12.8%) was similar to the aggregate LTFU estimate at 1 year from the meta-analysis (11.6%). Both of these estimates are hard to compare to previous systematic reviews of studies published prior to 2011, again, because of varying definitions and methodologies. Two large systematic reviews of studies published from lower-income countries estimated ± 20% LTFU in the first 6 months^3^ and 11% at 36 months,^5^ respectively. Two South African studies, both of which reviewed data from ± 2002 to 2007, estimated 13% LTFU at 1 year,^4^ and 18.7% at a median of 2.4 years,^6^ respectively. It is also important to note that the studies included in this review were conducted during years when the earlier South African ART guidelines were in place; the 2010 and 2013 guidelines utilised CD4 threshold for ART initiation of 200 and 350, respectively. The 2015 guidelines expanded ART to a CD4 threshold of 500, and more recently, universal test and treat has been adopted nationally. In more recent studies, Bock et al.^7^ reported 26% LTFU at 2 years in three South African public sector clinics through 2016; Grimsrud et al. reported 17% LTFU at 2 years in a similar patient population.^8^ While overall studies are reporting higher rates of LTFU as the ART thresholds have changed, there is still not a widely adopted definition and standardisation of measurement of LTFU. It is therefore hard to draw definitive conclusions about trends of LTFU rates over time.

Previous studies have examined the need for standardisation of a LTFU definition in ART programmes in sub-Saharan Africa, citing methodological concerns and a range of outcomes depending on the definition used.^82,83,84,85^ Indeed, there have been a variety of study definitions with a vast diversity of LTFU estimates. A review from 2007 of ART programmes in sub-Saharan Africa estimated 20% attrition at 6 months and between 25% and 75% at 2 years depending on the estimation method used.^86^ A subsequent review of studies from 2007 to 2009 estimated 29.5% attrition (death or LTFU) at 3 years; 59% of these patients were LTFU.^87^ A larger review by the same authors subsequently estimated 35% attrition in Africa by 36 months.^88^ In South Africa, a systematic review from 2014 estimated approximately 33% attrition by 4 years on ART using studies published between 2008 and 2013.^2^

Chi et al.^10^ performed a meta-analysis that included patients from 19 countries and 111 health facilities, concluding that a standard definition of 180 days since last clinic visit was most accurate in determining actual loss from care. Grimsrud et al.^11^ examined the impact of using different definitions of loss to follow-up on programme outcomes using data from the International Epidemiological Databases to Evaluate AIDS-Southern Africa, finding that utilising different definitions led to significantly different estimates of those LTFU, making it impossible to effectively compare rates from different programmes if the same definition is not used; these authors also recommended a standard definition of 180 days since last clinic visit. We support this standardised definition.

A key limitation of this study was that despite including 67 studies that met our inclusion criteria, there was not large heterogeneity in terms of study locations. Many of them were conducted in the same study site and often had overlapping dates. There were a disproportionate number of studies (two-thirds) in urban areas, primarily in Gauteng and Western Cape provinces. Fifteen (22%) of them came from a single clinic in Johannesburg (Themba Lethu). This overlap led to a significant decrease in the number of studies we could include in our meta-analysis and therefore reduced the likelihood we could find significant statistical differences in LTFU. For instance, we did not find that variation in LTFU definition impacted overall LTFU estimates at 1 year in our meta-analysis, and this is likely because of several reasons. Firstly, the small sample size of the analysis; once estimates were matched for definition and overlapping cohorts were removed, the sample size was relatively small. Similarly, larger estimates of LTFU are notable in smaller cohorts likely because of outlier effects. Secondly, there was a lack of standardisation of estimation methodologies for LTFU and mortality including length of follow-up time. Thirdly, inclusion of paediatric cohorts likely also played a role in the observed variation. For instance, paediatric patients may be more likely to be retained in care given that they have caregivers. Additionally, pregnant patients may be more likely to be lost to follow-up following childbirth, which has been demonstrated in several studies. This may be for a variety of reasons, including lifestyle changes postpartum as well as changing motivations after preventing HIV transmission to their infants.^89,90,91^ Indeed, the differences in aggregate LTFU estimates at 1 year were different between adult versus paediatric versus pregnancy cohorts and largely follow this trend: pregnancy cohorts had higher LTFU (33.0%), and paediatric lower LTFU (7.5%) than adult cohorts (11.6%). A final limitation was that six randomised controlled studies were included, of which some of the interventions were designed to impact adherence and LTFU, which therefore could have biased the meta-analysis estimates.

We likely underestimated and/or misrepresented true estimates of LTFU at 5 years in our meta-analysis because of including only two non-representative cohorts in our estimate after standardisation. However, both estimated LTFU at 5 years to be > 1 in five patients. Fatti et al.^22^ defined LTFU as 187 days without a clinic visit and did not include those who had left care and returned later. Grimsrud et al.^81^ similarly defined LTFU as 6 months without a clinic visit and also did not include patients who had left care and returned later. Despite being high crude rates of LTFU, these are lower than estimated by large systematic reviews as described above.

In conclusion, going forward in South Africa, our data suggest that it would be helpful for policy-makers to recommend and programme managers to put into practice a system in which the definition of LTFU or ‘default’ from care is standardised across South African ART programmes. Such standardisation would not only aid in comparing outcomes across clinics and across the country, especially at defined timeframes, but also in planning broadly applicable interventions for patient retention. Ideally, data from clinics could be monitored in real time using a standardised definition, with an actionable reporting system in place to identify patients who require re-engagement, or clinics that need interventions to improve patient retention. Additionally, tracing patients after they are LTFU may improve outcomes and lower LTFU rates, as many ART patients are mobile and receiving care at more than one clinic, and/or transfers to other clinics may not be sufficiently documented in current data systems.^12,92,93,94^ Already a three-tiered monitoring system exists in the Western Cape Province that aggregates paper and electronic systems into a single database for reporting purposes^95^; the ideal or goal is to scale this up to a national level and transition to an electronic medical record as resources allow. We hope that our data may be useful to South African ART programmes in advancing these broader goals of improving ART retention for patients across South Africa.

## Appendix 1

### PubMed search strategy

1. (((((((((((((((((“HIV Infections”[Mesh]) OR “HIV”[Mesh]) OR HIV*) OR hiv-1) OR hiv-2) OR hiv1) OR hiv2) OR hiv infect*) OR human immunodeficiency virus) OR human immune deficiency virus) OR human immuno-deficiency virus) OR human immune-deficiency virus) OR (((human immune*) AND (deficiency virus)))) OR acquired immunodeficiency syndrome*) OR acquired immune deficiency syndrome*) OR acquired immuno-deficiency syndrome) OR acquired immune-deficiency syndrome) OR (((acquired immun*) AND (deficiency syndrome)))
2. ((((((((((“anti-HIV agents”[MeSH]) OR “antiretroviral therapy, highly active”[MeSH]) OR HAART) OR cART) OR ART) OR antiretroviral) OR anti-retroviral) OR anti-viral) OR antiviral) OR antiviral therapy) OR ARV
3. ((((((((((((((((((((((((((“patient compliance”[MeSH]) OR “lost to follow-up”[MeSH]) OR “treatment outcome”[MeSH]) OR “treatment refusal”[MeSH]) OR “continuity of patient care”[MeSH]) OR retention) OR nonadherence) OR non-adherence) OR adherence) OR noncompliance) OR non-compliance) OR follow-up) OR patient monitoring) OR attrition) OR patient elopement) OR retain*) OR (((loss*) and “follow-up”))) OR LTFU) OR “loss to care”) OR “lost to follow-up”) OR “loss to follow-up”) OR “lost to care”) OR “loss to program*”) OR “lost to program*”) OR default*) OR engage*) OR disengage*
4. (“South African”) OR “South Africa*”
5. #1 AND #2 AND #3 AND #4

## Appendix 2

**TABLE 1-A2:** Modified Newcastle-Ottawa Scale.

Question	Options
Is the exposed cohort representative of the clinic population?	Yes, definitely representative of the clinic population Yes, probably representative of the clinic population (exclusions based on convenience or missing data were <10% of total eligible population) No; selected group of users Unclear; or no description of the derivation of the cohort
Was starting ART ascertained using reliable data?	Yes; secure medical records No Unclear; or no description
Did study ascertain and record death?	Yes No
Was assessment of outcome independent?	Yes, record linkage No Unclear, or no description
Was follow-up long enough for outcomes to occur?	Yes, definitely; Median follow-up > 3 years Yes, probably; Median follow-up 1–3 years No; Median follow-up < 1 year Unclear length of follow-up
Was missing data minimal and accounted for? (e.g. how many patients could be classified as in care or LTFU?)	Yes, definitely; complete data – all subjects/records accounted for Yes, probably; missing data unlikely to introduce bias – small number lost – < 10% related to data on LTFU or death outcomes (or description provided of those missing) No; missing data > 10% and or no description of those missing Unclear; no statement

*Source*: Ottawa Hospital Research Institute. The Newcastle-Ottawa Scale (NOS) for assessing the quality of nonrandomised studies in meta-analyses. 2019. [cited 2019 Sep 2]. Available from: http://www.ohri.ca/programs/clinical_epidemiology/oxford.asp

LTFU, loss to follow-up; ART, antiretroviral therapy.
